# A pilot study on DNA methylation changes for non‐invasive molecular diagnostics in heart failure

**DOI:** 10.1002/ehf2.15402

**Published:** 2025-09-02

**Authors:** Giuditta Benincasa, Francesco Cacciatore, Mark E. Pepin, Francesco Curcio, Rosaria Chiappetti, Adam R. Wende, Enrico Coscioni, Claudio Napoli

**Affiliations:** ^1^ Department of Advanced Medical and Surgical Sciences (DAMSS) University of Campania ‘Luigi Vanvitelli’ Naples Italy; ^2^ Department of Translational Medical Sciences University of Naples ‘Federico II’ Naples Italy; ^3^ Stanford Cardiovascular Institute Stanford University School of Medicine Stanford California USA; ^4^ Division of Cardiovascular Medicine, Department of Medicine Stanford University School of Medicine Stanford California USA; ^5^ Division of Molecular and Cellular Pathology, Department of Pathology University of Alabama at Birmingham Birmingham Alabama USA; ^6^ Division of Cardiac Surgery AOU San Giovanni di Dio e Ruggi d'Aragona Salerno Italy

**Keywords:** biomarkers, CD4^+^ T cells, DNA methylation, heart failure, interactome, network medicine, RRBS

## Abstract

**Aims:**

The current therapeutic approach to ischaemic (IsHF) and non‐ischaemic (NIsHF) heart failure (HF) mainly overlooks the underlying aetiology owing to a lack knowledge of the differential molecular pathways that contribute to HF with reduced ejection fraction (HFrEF). Alterations in myocardial DNA methylation levels have been identified as potential biomarkers for HF irrespective of its aetiology. Due to the limited availability of cardiac tissues in clinics, our goal is to determine if DNA methylation changes in circulating CD4^+^ T lymphocytes, which are strongly involved in left ventricle remodelling, can help in differentiating IsHF and NIsHF causes among patients with HFrEF and if DNA methylation levels associate with key clinical features.

**Methods and results:**

We performed a post hoc network‐oriented analysis of the original PRESMET clinical trial dataset (NCT05475028). Integrating epigenomic data obtained with the high‐resolution reduced representation bisulfite sequencing (RRBS) platform and the left‐ventricle interactome (protein–protein interaction map) we identified six differentially methylated CpG positions (DMPs), which were able to distinguish IsHF (*n* = 8) versus NIsHF (*n* = 4) patients with an area under the curve (AUC) > 0.8. Network‐oriented DMPs were significantly hypomethylated in IsHF versus NIsHF and annotated to six genes, namely, cytoskeleton‐associated protein 4 (*CKAP4*), carnitine palmitoyltransferase 1A (*CPT1A*), eukaryotic translation initiation factor 2 subunit beta (*EIF2S2*), spectrin beta (*SPTB*), synaptotagmin 6 (*SYT6*) and RAB11 family interacting protein 1 (*RAB11FIP1*) (*P* < 0.05). We found that the CD4^+^ T cell hypomethylation of *EIF2S2* gene significantly correlated with VO_2_ max (*ρ* = 0.73, *P* = 0.04)*,* hypomethylation of *RAB11FIP1* significantly correlated with MECKI score (*ρ* = −0.85, *P* = 0.001), whereas *SPTB* significantly correlated with VO_2_ max (*ρ* = 0.73, *P* = 0.04), left ventricle mass index (*ρ* = −0.91, *P* = 0.005) and left ventricular ejection fraction (*ρ* = 0.83, *P* = 0.01) in IsHF patients.

**Conclusions:**

We demonstrate that circulating CD4^+^ T cell‐specific methylation levels of network‐oriented *CKAP4*, *CPT1A*, *EIF2S2*, *SPTB*, *SYT6* and *RAB11FIP1* genes can distinguish between IsHF and NIsHF. In particular, hypomethylation of *EIF2S2*, *SPTB* and *RAB11FIP1* genes is significantly correlated with key clinical features in isHF patients, highlighting its potential to enhance personalized prognosis for HFrEF patients.

## Graphical abstract

A liquid biopsy approach integrating the high‐resolution RRBS DNA methylation platform and network‐oriented strategy identified the hypomethylation of *CPTA1*, *CKAP4*, *SPTB*, *SYT6*, *EIF2S2*, and *RAB11FIP1* genes as useful biomarkers in distinguishing between ischaemic and non‐ischaemic HF aetiologies. Moreover, the epigenetic‐sensitive changes strongly correlated with clinical prognostic indices including MECKI score, VO_2_ max, LVMI and LVEF. This innovative approach paves the way for tailored therapeutic strategies and improved risk stratification in support of the established HF work‐up.

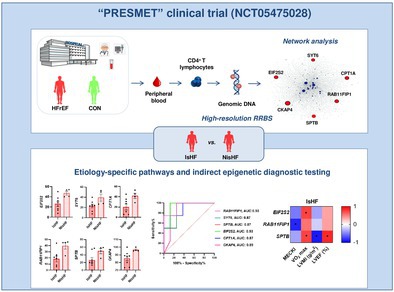

## Introduction

Heart failure (HF) with reduced ejection fraction (HFrEF) represents a significant clinical and epidemiological burden, commonly emerging as a ‘final common pathway’ for various cardiac diseases, despite their distinct aetiologies.^1^ Among these, coronary heart disease (CHD) is a predominant contributor to HFrEF.^2^ Currently, the therapeutic approach to ischaemic (IsHF) and non‐ischaemic (NIsHF) HF largely overlooks the underlying aetiology, which reflects our incomplete understanding of the diverse molecular pathways that contribute to HFrEF.^3^, ^4^ This gap in knowledge underscores the need for more precise molecular phenotyping of HF, which is currently hindered by limited access to myocardial tissue and the variability of circulating biomarkers.^5^ Consequently, there is a growing interest in a ‘liquid biopsy’ approach that captures the pathogenic epigenetic signatures, aiming to enhance the precision of HF medicine and personalized therapeutic strategies.^6^ Despite numerous studies identifying distinct DNA methylation patterns in cardiac tissue and peripheral blood of HF patients,^7^, ^8^, ^9^, ^10^, ^11^, ^12^, ^13^, ^14^, ^15^ the clinical implications of these findings are yet to be fully explored. Our group has extensively studied circulating CD4^+^ T cell‐specific DNA methylation changes in patients with atrial fibrillation,^16^ pulmonary arterial hypertension,^17^ acute coronary syndrome^18^ and increasing hyperglycaemia,^19^ in which we identified novel epigenetic targets of potential clinical use. Because CD4^+^ T lymphocytes play a key role in left‐ventricular remodelling during IsHF,^20^, ^21^ our goal is to determine if high‐resolution epigenetic testing can discern between IsHF and NIsHF, potentially leading to more targeted and effective treatments.

## Methods

### Study population

We conducted a post hoc analysis of the PRESMET study (NCT05475028), an observational clinical trial designed to profile differential methylation in patients with HFrEF as compared with those with preserved ejection fraction (HFpEF).^22^ For the purposes of the current study, we re‐purposed this dataset to exclusively include HFrEF patients who underwent coronary angiography at diagnosis to determine the aetiology.

### Liquid‐based assays, high‐resolution RRBS platform and bioinformatic pipeline

As previously described,^16^, ^17^, ^18^, ^19^, ^22^ we collected approximately 8 mL of peripheral blood biospecimens for each study participant. Peripheral blood mononuclear cells (PBMCs) were isolated on a Ficoll gradient using Histopaque®‐1077 with subsequent purification for CD4^+^ T cells via EasySep™ Human CD4^+^ T Cell Isolation Kit (Stem Cell), performed in accordance with the manufacturer's instructions. Genomic DNA (gDNA) was extracted using DNeasy Blood & Tissue Kit (Qiagen) and stored at −80°C until sequencing analysis. High‐resolution RRBS was performed at the Genomix4Life S.r.l. (Salerno, Italy) using a protocol that has been previously described.^23^ We briefly summarize the details of the PRESMET study bioinformatic analysis.^22^ The raw sequence files generated (fastq files) underwent quality control analysis using FastQC (0.11.9) before and after adapter trimming in order to ensure PHRED<30 via TrimGalore (0.4.4). The bisulfite‐reduced and sequenced reads were then aligned to CT‐ and GA‐converted human hg38 (GRCh38.p12) genome assemblies using Bismark to quantify relative alignment of methylated and unmethylated CpGs, respectively. The alignment yielded a mapping efficiency of 48.9% ± 2.7% (9.8 ± 2.5 million) paired‐end reads after quality trimming and alignment. To ensure data analysis was not biased by coverage or PCR amplification, reads were removed with counts <5 and/or % methylation >99.9%; doing so removed ~0.10% of reads per sample. Differential DNA methylation was computed using a 1‐base window to exploit the regional CpG methylation analysis afforded by RRBS using the R package methylKit (1.8.0). Originally, we designed the differential DNA methylation analysis of CD4^+^ T cells using a machine‐learning model to identify significant associations in a discovery cohort which were validated using an independent cohort.^22^ To identify reproducible aetiology‐dependent features of differential DNA methylation, we obtained age‐ and comorbidity‐adjusted samples from both discovery and validation cohorts in HFrEF. Samples were analysed separately to avoid confounding batch effects. Then, we merged the two lists of annotated DMPs deriving from both discovery and validation datasets by genomic location. Statistical significance of DMPs was assumed based on an over‐correction adjusted *χ* ^2^ test. To adjust *P* values for multiple testing, a post hoc sliding linear model method was applied.

### Network analysis

We used the list of HFrEF‐specific DMPs which were common to IsHF and NIsHF patients^22^ as input for NetworkAnalyst 3.0 tool (http:// www.networkanalyst.ca) to build the left ventricle‐specific protein–protein interaction (PPI) network (or interactome). The interactome is a map of cellular‐specific PPIs, which guides biological function in a specific spatio‐temporal manner. The interactome represents a useful tool to identify the ‘disease‐specific module’, which potentially includes candidate genes and novel drug targets.^24^, ^25^, ^26^ Gene set enrichment analysis was performed by using the Enrich database (http://amp.pharm.mssm.edu/Enrichr) via Reactome Pathways 2024.

### Statistical analysis

All statistical analyses were performed using Graph Pad PRISM Version 8.0.2 software (GraphPad Software, CA, USA). The receiver operating characteristic (ROC) analysis was performed to identify DMPs and annotated genes that discriminated IsHF versus NIsHF. Spearman's analysis was used to determine correlations between DNA methylation levels and clinical parameters. Statistical significance was assumed at *P* < 0.05.

## Results

### Clinical characteristics of HFrEF patients

Demographic and clinical characteristics of HFrEF patients are summarized in *Table* *1*. For large‐scale DNA methylation study, we analysed eight IsHF patients (1F, 7M) with a mean age of 60.13 ± 11.58 years and four NIsHF patients (1F, 3M) with a mean age of 56.50 ± 6.95 years. Patients had an equal distribution of body mass index (BMI), comorbidities including hypertension, type 2 diabetes, atrial fibrillation/flutter and previous hospitalization. The mean New York Heart Association (NYHA) class was 2.3 ± 0.7 and 1.5 ± 0.5 in IsHF and NIsHF, respectively. All patients were under optimized therapy at the maximally tolerated dosage of ARNI, B‐blockers, SGLT2i and statins.

**Table 1:** Demographic and clinical characteristics of HFrEF patients stratified by ischaemic and non‐ischaemic aetiology.

	IsHF (*n* = 8)	NIsHF (*n* = 4)	*P*
Demographics			
Age (years)	60.13 ± 11.58	56.50 ± 6.95	0.582
Female sex, *n* (%)	1 (12.5)	1 (25.0)	0.954
BMI (kg/m^2^)	29.86 ± 4.61	26.85 ± 4.56	0.310
Medical history, *n* (%)			
HTN	5 (62.5)	2 (50.0)	0.679
T2D	5 (62.5)	1 (25.0)	0.221
AF/flutter	4 (50.0)	3 (75.0)	0.408
CRT‐D	1 (12.5)	3 (75.0)	0.030
ICD	6 (75.0)	1 (25.0)	0.908
CABG	3 (37.5)	0 (0.0)	0.157
Previous HF hospitalization	5 (62.5)	1 (25.0)	0.222
NYHA class	2.3 ± 0.7	1.5 ± 0.5	0.160
Cardiac function			
LVEF, %	31.50 ± 5.75	35.25 ± 3.77	0.270
LVMI, g/m^2^	121.65 ± 21.06	114.00 ± 23.57	0.614
LAVI, mL/m^2^	46.52 ± 19.31	46.52 ± 19.31	0.608
RWT	0.27 ± 0.02	0.26 ± 0.03	0.591
E/e′ ratio	10.03 ± 4.53	9.06 ± 2.01	0.730
E/A ratio	1.51 ± 1.29	1.09 ± 0.43	0.680
Functional parameters			
6MWT (m)	308.75 ± 118.40	308.75 ± 118.40	0.162
VO_2_ max (mL/kg/min)	12.77 ± 3.48	16.17 ± 3.39	0.140
VE/VCO_2_ (slope)	36.43 ± 12.94	30.85 ± 3.85	0.428
Prognostic score			
MECKI score (2 years)	11.7 ± 13.8	3.2 ± 0.8	0.127
SEATTLE score (1 year)	96.47 ± 1.97	98.20 ± 0.30	0.178
SEATTLE score (5 years)	83.83 ± 8.46	91.36 ± 1.60	0.173
Laboratory values			
NT‐proBNP (pg/mL)	1300.83 ± 1887.18	1027.02 ± 1410.93	0.804
HbA1C (%)	6.37 ± 1.04	6.10 ± 1.49	0.734
Creatinine (mg/dL)	1.40 ± 0.43	1.09 ± 0.25	0.269
eGFR (MDRD ml/min)	59.27 ± 20.59	67.13 ± 7.73	0.546
Uric acid (mg/dL)	4.68 ± 1.35	4.36 ± 1.30	0.488
N (mg/dL)	60.25 ± 23.27	41.0 ± 5.29	0.202
K (mmol/L)	4.61 ± 0.59	4.76 ± 0.51	0.700
Na (mmol/L)	141.75 ± 6.45	142.33 ± 2.30	0.835
Haemoglobin (g/dL)	14.30 ± 1.89	13.60 ± 2.35	0.619
Total bilirubin (mg/dL)	0.60 ± 0.23	1.07 ± 0.53	0.074

*Note*: Data are expressed as absolute number (%) or mean ± *SD*.

Abbreviations: AF, atrial fibrillation; BMI, body mass index; CABG, coronary artery bypass graft surgery; CRT‐D, cardiac resynchronization therapy with defibrillator; eGFR, estimated glomerular filtration rate; HTN, hypertension; ICD, implantable cardioverter defibrillator; IsHF, ischaemic heart failure; LAVI, left atrial volume index; LVEF, left ventricle ejection fraction; LVMI, left ventricle mass index; MDRD, modification of diet in renal disease; NIsHF, non‐ischaemic heart failure; RWT, relative wall thickness; 6MWT, 6 min walking distance; T2D, type 2 diabetes.

### DNA methylomics and network prioritization of candidate genes

We identified 133 HFrEF‐specific DMPs located within coding and non‐coding regions, defined as differentially methylated genes (DMGs), which were shared between IsHF and NIsHF patients. Then, we built an HFrEF‐specific interactome using the NetworkAnalyst 3.0 tool (http://www.networkanalyst.ca) and selecting the left ventricle‐specific PPI network from the DifferentialNet database. Construction of a HFrEF‐specific interactome identified 62 ‘nodal genes’, which represent those proteins with the highest number of interactions with proteins which have already been implicated in ‘Left ventricle (LV) function’ supporting their biological role in disease pathogenesis. Each DMG had at least five independent associations with the LV functionally‐related proteins of the human interactome (*Table* S1). Functional analysis revealed a significant enrichment in pathways involved in signal transduction, cellular responses to stimuli, cytokine signalling in immune system, chromatin‐modifying enzymes and adipogenesis (*Table* S2).

### DNA methylation diagnostic performance

**Figure 1:**
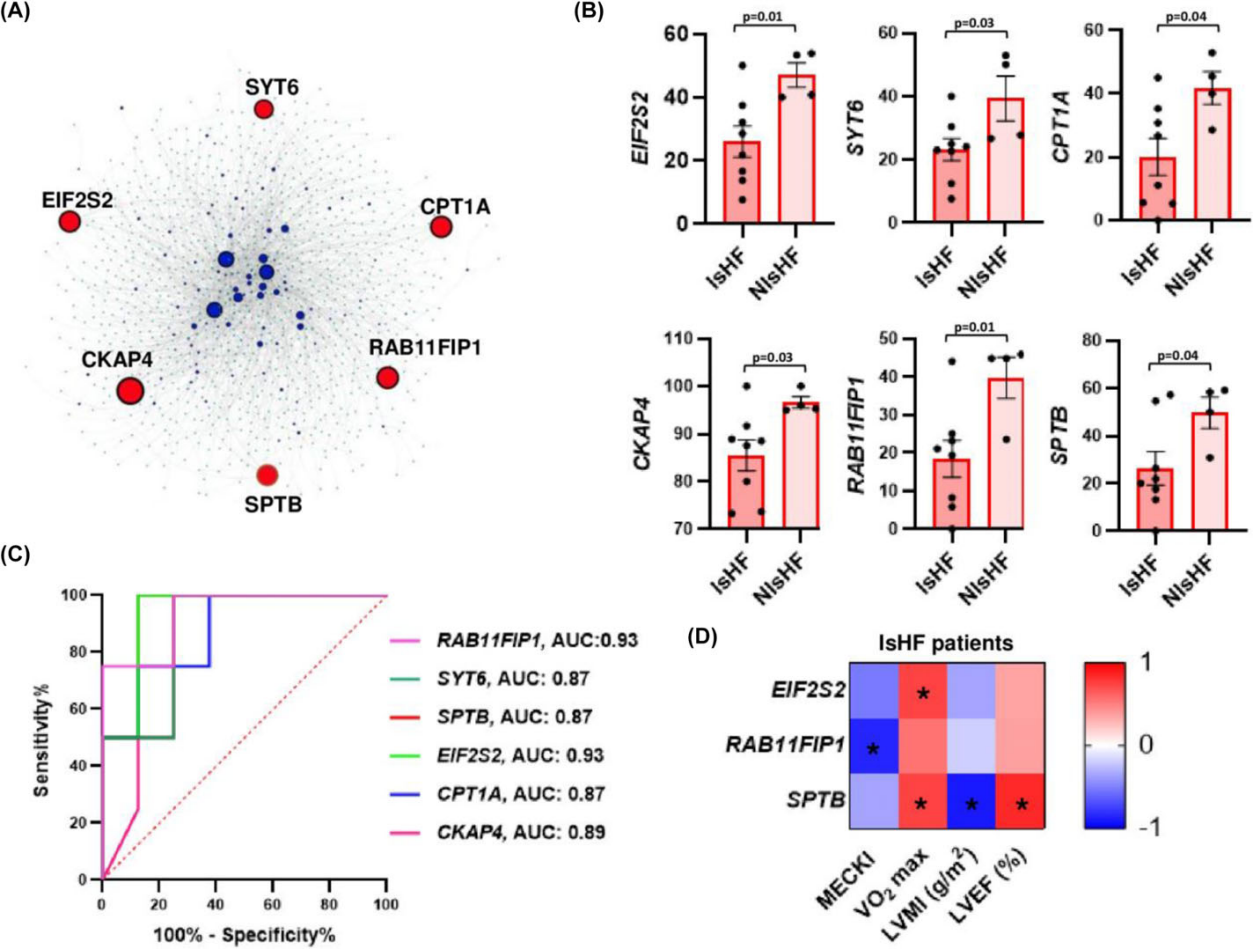
Clinical potential of DNA methylation changes in IsHF versus NIsHF. (A) The left‐ventricle interactome shows six nodal genes (DMGs) able to distinguish IsHF from NIsHF patients, which are represented by red circles. Additional interacting genes/proteins are represented by blue circles. The protein–protein interactions (links) are represented by grey edges among circles. The size of circles is scaled according to the number of degree for each nodal gene (original source: NetworkAnalyst 3.0). (B) Dot plots show that *EIF2S2*, *SYT6*, *CPTA1*, *CKAP4*, *RAB11FIP* and *SPTB* were significantly hypomethylated in 8 IsHF versus 4 NIsHF patients. (C) Receiver operating characteristic (ROC) curve analysis shows that methylation levels of the *CPTA1*, *CKAP4*, *SPTB*, *SYT6*, *EIF2S2* and *RAB11FIP1* genes have an area under the curve (AUC) > 80% supporting their diagnostic potential in discriminating IsHF versus NIsHF patients (*P* < 0.05); (D) Heatmap shows significant correlations between reduced methylation levels of *EIF2S2* and VO_2_ max, reduced methylation levels of *RAB11FIP1* gene and MECKI score, as well as reduced methylation levels of *SPTB* and VO_2_ max, LVMI and LVEF. Positive correlations are showed in red and negative ones in blue. *P < 0.05. *CKAP4*, cytoskeleton‐associated protein 4; *CPTA1*, carnitine palmitoyltransferase 1A; *EIF2S2*, eukaryotic translation initiation factor 2 subunit beta; IsHF, ischaemic heart failure; LVEF, left ventricle ejection fraction; LVMI, left ventricle mass index; NIsHF, non‐ischaemic heart failure; *RAB11FIP1*, RAB11 family interacting protein 1; *SPTB*, spectrin beta; *SYT6*, Synaptotagmin 6.

We identified network‐oriented DMGs able to distinguish IsHF and NIsHF patients (*Table* *2* and *Figure* *1A*). Our analysis revealed that methylation levels of six DMGs significantly discriminated IsHF versus NIsHF patients (Mann–Whitney test, *P* < 0.05): cytoskeleton‐associated protein 4 (*CKAP4*), carnitine palmitoyltransferase 1A (*CPT1A*), eukaryotic translation initiation factor 2 subunit beta (*EIF2S2*), spectrin beta (*SPTB*), synaptotagmin 6 (*SYT6*) and RAB11 family interacting protein 1 (*RAB11FIP1*) (*Figure* *1B*). To approximate their prediction of HFrEF aetiology, we used ROC curve analysis to show that methylation levels of six DMPs (*Table* *2*) which annotated to *CKAP4* (AUC:0.89, *P* = 0.03), *CPT1A* (AUC:0.87, *P* = 0.04), *EIF2S2* (AUC:0.93, *P* = 0.01), *SPTB* (AUC:0.87, *P* = 0.04), *SYT6* (AUC:0.87, *P* = 0.04) and *RAB11FIP1* (AUC:0.93, *P* = 0.01) significantly discriminated IsHF from NIsHF patients with an AUC > 80% (*Figure* *1C*). We combined functional patient‐specific testing to show that *EIF2S2* significantly correlated with VO_2_ max (*ρ* = 0.73, *P* = 0.04), whereas *SPTB* significantly correlated with VO_2_ max (*ρ* = 0.73, *P* = 0.04), left ventricle mass index (LVMI) (*ρ* = −0.91, *P* = 0.005) and LVEF (*ρ* = 0.83, *P* = 0.01), and *RAB11FIP1* significantly correlated with MECKI score (*ρ* = −0.85, *P* = 0.001) in IsHF patients (*Figure* *1D*).

**Table 2:** Diagnostic performance of network‐oriented DMGs.

DMP	DMG	Genomic position	AUC	*P* value
chr12: 106260926	*CKAP4*	Intron	0.89	0.03
chr11: 68839227	*CPT1A*	Intron; CpG shore	0.87	0.04
chr20: 34111635	*EIF2S2*	Intron; CpG island	0.93	0.01
chr14: 64823346	*SPTB*	Promoter	0.87	0.04
chr1: 114154901	*SYT6*	1–5 kb; CpG shore	0.87	0.04
chr8: 37899416	*RAB11FIP1*	5′UTRs; Cpg island	0.93	0.01

## Discussion

This study represents the first aetiology‐specific analysis aimed at assessing the diagnostic performance of RRBS‐derived circulating DNA methylation changes in IsHF versus NIsHF. We identified *CKAP4*, *CPT1A*, *EIF2S2*, *SPTB*, *SYT6* and *RAB11FIP1* as ‘indirect’ methylation‐sensitive loci, which could enhance network‐oriented precision medicine strategies for HFrEF.

Until now, the genes *CPT1A*, *EIF2S2*, *SPTB* and *SYT6* have not been linked to the pathogenesis of HF. However, previous clinical studies have demonstrated that the RNA‐binding protein CKAP4 plays a role in regulating cardiac function and pathological remodelling in enlarged atrial myocardium.^27^ Single‐cell RNA sequencing of adult cardiac tissues has identified the *CKAP4* gene as a novel marker for activated fibroblasts, showing a positive correlation with established myofibroblast markers in both mouse and human heart tissues.^28^ Interestingly, prior research has shown that the *RAB11FIP1* gene is both hypermethylated and transcriptionally upregulated in cardiac tissues of patients with IsHF compared with NIsHF, further validating our approach of using indirect epigenetic testing to elucidate myocardial pathophysiology. Building on these insights, the application of these methylation‐sensitive markers in diagnosing the origin of HF at clinical presentation becomes particularly compelling.^7^ Often, symptoms of HF manifest in ischaemic patients as the initial clinical presentation, even without a documented ischaemic event in their medical history. This early symptomatology, which may precede clear clinical indicators of coronary artery disease, could play a role for a diagnostic intervention.

Moreover, the ability to identify early epigenetic signatures linked to the inheritance of cardiovascular risk may help to stratify individuals before cardiac symptoms occur. Such stratification could enable targeted monitoring and preventative strategies for at‐risk populations, potentially delaying or preventing the onset of HF. By leveraging these epigenetic markers, clinicians could refine their diagnostic processes, not only enhancing the accuracy of their initial assessments but also customizing preventative care tailored to the molecular profile indicative of future cardiovascular events.

Interestingly, the methylation levels of the *RAB11FIP1* gene significantly predict the MECKI score, which allows for an accurate evaluation of HF risk using only six variables. These variables include assessments of exercise capacity (peak oxygen uptake and ventilation/CO_2_ production slope), blood samples (haemoglobin, sodium and renal function) and echocardiography [left ventricular ejection fraction (LVEF)].^29^ Our findings suggest that integrating epigenetic fingerprinting with the functional MECKI score could enhance the predictive accuracy of HF risk stratification. This hypothesis warrants further longitudinal studies to determine whether such integration improves the resolution of earlier predictive models for HF risk.^30^

The current study has several limitations. First, it is an observational, post hoc analysis of the original PRESMET study^22^ including a total of 46 study participants of which 12 patients had a diagnosis of HFrEF. Data from HFrEF patients were used to obtain preliminary evidence of the usefulness of circulating methylomes in distinguishing ischaemic from non‐ischaemic causes of HF for the first time.^22^ We recognize that the small sample size warrants caution in interpretation of results because of the lowest robustness in supporting the validity of the statistical analyses, especially for the ROC and correlation analyses (e.g., VO_2_ max, LVEF) which are intriguing but remain exploratory. Clinical studies enrolling larger cohort of patients are ongoing. Furthermore, the small sample size lacks the statistical power to test the performance of epigenetic biomarkers across various sub‐populations of HF patients that may have clinical relevance, such as those defined by gender, race, socioeconomic status or medications. We highlight that all HFrEF patients were under optimized therapy at the maximally tolerated dosage of ARNI, B‐blockers, SGLT2i and statins. Thus, we can exclude any confounding effect between methylation patterns and medications in comparing isHF versus NiHF. Another limitation is that the unavailability of sufficient genomic DNA biospecimens did not allow us to validate the epigenetic signatures using an alternative platform, such as the pyrosequencing, to confirm the RRBS results.

However, to counterbalance the small sample size, that is common for hypothesis‐generating studies, we designed the analysis of CD4^+^ T cell‐specific DNA methylation using three key strategies: (1) the high‐resolution RRBS platform which is the gold‐standard for measuring genome‐wide DNA methylation levels at single‐cytosine level in peripheral blood, a feature that allows us to obtain significant evidence of epigenetic signatures also in pilot studies^16^, ^17^, ^18^, ^19^, ^22^; (2) a two‐step machine‐learning approach to first identify associations in a discovery cohort (*n* = 7 HFrEF), followed by validation using an independent cohort (*n* = 5 HFrEF).^22^ This design reduces the risk of overfitting and false positives, as the identified associations are subsequently tested. Of note, the two‐phase approach allows us to verify that observed associations are not artefacts due to sample‐specific variability but represent more generalizable findings; (3) advanced network‐oriented bioinformatic strategies based on the interactome which is one of the most useful tool to indentify novel disease genes with clinical potential.^24^, ^25^, ^26^

Additionally, the cross‐sectional nature of the study precludes the ability to establish causal relationships between changes in methylation levels and clinical outcome. This limitation underscores the need for longitudinal studies on adequate number of patients to more definitively ascertain the dynamics and implications of epigenetic modifications in HF. Moreover, further studies should evaluate possible changes in 5‐hydroxymethylcytosine (5hmC) which is an important epigenetic mark that regulates gene expression^31^ as well as the possibility that identified methylation markers may be also detected in the methylome of circulating cell‐free DNA (ccf‐DNA). This because ccf‐DNA represents short DNA fragments wrapped around nucleosomes arising from the process of cell death and, importantly, may retain the same epigenetic information as the tissue of origin mirroring cardiac‐specific epigenetic abnormalities in HF patients.^32^

## Conclusions

In conclusion, this study highlights the promising potential of RRBS‐derived DNA methylation profiling in distinguishing between ischaemic and non‐ischaemic HF aetiologies. Our findings identify specific methylation‐sensitive loci that not only differentiate between HF types but also correlate strongly with clinical prognostic indices, as previously showed in major cardiovascular diseases.^16^, ^17^, ^18^, ^19^, ^22^, ^33^ This emphasizes their potential role in elucidating distinct pathophysiological profiles, paving the way for tailored therapeutic approaches and improved risk stratification in the future.

## Funding

This work was supported by the Ministry of University and Research (MUR) by the following research grants: PRIN2020XMLP45 and PNRR‐AGE‐IT‐PE0000015 (P.I. Prof. Claudio Napoli).

## Conflict of interest statement

The authors declare that they have no conflict of interest.

## Supporting information


**Table S1.** Hubs.
**Table S2.** Top five significant pathways.
