# Supplementary material for: A pilot study on DNA methylation changes for non‐invasive molecular diagnostics in heart failure
Source: ESC Heart Fail. 2025 Sep 2;12(6):4513–20. doi: 10.1002/ehf2.15402 (PMC12719838; doi:10.1002/ehf2.15402)
Supplement: Supplementary file 1 — Table S1. Hubs. Table S2. Top five significant pathways. [file EHF2-12-4513-s001.docx]

Supplementary Table 1. Hubs

| Gene | Function | Degree |
| --- | --- | --- |
| *HIST1H4E* | Interacting protein | 123 |
| *STK4* | DMG | 118 |
| *ERG* | DMG | 105 |
| *DAPK1* | DMG | 47 |
| *ING5* | DMG | 42 |
| *FGFR3* | DMG | 38 |
| *ECHS1* | DMG | 34 |
| *PASK* | DMG | 32 |
| *SRSF5* | DMG | 28 |
| *PLEC* | DMG | 27 |
| *KCND3* | DMG | 27 |
| *MARK2* | DMG | 24 |
| *THRB* | DMG | 22 |
| *VPRBP* | Interacting protein | 22 |
| *MED17* | DMG | 19 |
| *CKAP4* | DMG | 18 |
| *COX17* | DMG | 16 |
| *MECOM* | DMG | 16 |
| *DUSP1* | DMG | 16 |
| *XPO7* | DMG | 14 |
| *CPT1A* | DMG | 14 |
| *MRFAP1* | DMG | 14 |
| *EIF2S2* | DMG | 13 |
| *DOCK5* | DMG | 13 |
| *MEF2C* | DMG | 12 |
| *FOSL2* | DMG | 12 |
| *SPTB* | DMG | 12 |
| *SIRT7* | Interacting protein | 11 |
| *ASAP2* | DMG | 11 |
| *GCDH* | DMG | 11 |
| *MOV10* | Interacting protein | 10 |
| *ZC3H18* | DMG | 10 |
| *ATP6V1E1* | DMG | 9 |
| *NR2F6* | DMG | 9 |
| *DOCK9* | DMG | 9 |
| *GP1BB* | DMG | 9 |
| *NXF1* | Interacting protein | 8 |
| *NUFIP2* | DMG | 8 |
| *GOT1* | DMG | 8 |
| *COPS5* | Interacting protein | 7 |
| *MYC* | Interacting protein | 7 |
| *VPS4B* | DMG | 7 |
| *SYT6* | DMG | 7 |
| *IRAK3* | DMG | 7 |
| *CBX7* | DMG | 7 |
| *ELAVL1* | Interacting protein | 6 |
| *MUM1* | Interacting protein | 6 |
| *ASPM* | DMG | 6 |
| *NNT* | DMG | 6 |
| *INA* | DMG | 6 |
| *HELZ* | DMG | 6 |
| *GSTM4* | DMG | 6 |
| *CUL3* | Interacting protein | 5 |
| *YWHAZ* | Interacting protein | 5 |
| *CCDC8* | Interacting protein | 5 |
| *CUL7* | Interacting protein | 5 |
| *PAX5* | DMG | 5 |
| *RAI1* | DMG | 5 |
| *RAB11FIP1* | DMG | 5 |
| *SMTN* | DMG | 5 |
| *FBN3* | DMG | 5 |
| *TNFRSF11A* | DMG | 5 |

Supplementary Table 2. Top five significant pathways

| Pathway | p-value | Genes |
| --- | --- | --- |
| Signal Transduction | 4.386^e-41^ | ATF2;APP;TRRAP;HDAC11;SOX2;PSMD7;PPP2R1B;PPP2R1A;MYC;RPS6KA1;ATP6V1E1;JUNB;SOX4;MEF2C;PRKCI;CSNK2A2;PRKAB1;RUNX1;SRRM1;AR;CDC37;PARD3;KCTD13;MAML3;TP53;OPTN;PFN2;SHMT2;CTBP1;SH2D2A;TWF1;KLK3;IQGAP1;KLK2;IQGAP2;NLK;IQGAP3;RAD21;HIST1H3G;SFN;APOE;PLCG1;FADD;PCK1;STAT5A;STAT5B;JUN;CREBBP;RCC2;FN1;PPBP;ZWINT;MOV10;CYP26A1;HNRNPM;SNW1;HNRNPF;ID3;GRB2;HNRNPC;CALM1;EZH2;CDKN1A;SH3KBP1;CLTC;BMI1;IKBKB;TUBA1C;TUBA1B;XPO1;MECOM;SPTAN1;EMD;NDEL1;MYOG;NCOA2;SREBF1;CCT2;ANXA1;RING1;PARP1;DUSP1;RIPK2;PRMT1;NCOA3;GAB1;PTH2R;FOS;DUSP9;UCHL5;CNKSR1;KAT2B;KAT2A;CTTN;KIF2A;HNRNPH1;KIF2C;TFF1;PHLPP1;CENPA;EGFR;RELA;PPP2CA;TUBA3C;RXRA;PSMB1;TBL1X;CCT7;PAK3;RXRG;PAK2;MBD3;SUZ12;RANBP2;STAT1;USP9X;STAT3;DAB2IP;TUBB4B;MIB1;TJP1;TAOK1;NEDD4;ABI1;CTNNB1;GOPC;KBTBD7;LGR4;CD86;PLAT;FGF1;FGF2;FGF4;FGF5;CCND1;FGF9;RUVBL1;EP300;TLE4;TLE1;FBXW7;TPM3;TMOD3;WNT5A;TSC2;TSC1;TUBA4A;RNF41;CCNE1;HGS;SQSTM1;FKBP5;ATP6V1A;DDX5;VCP;CUL5;CUL3;PSEN2;CUL1;ARHGAP18;PHB;ADRB2;FOXO3;STK4;SAV1;STK3;SPTA1;SOCS3;SOCS1;UBB;UBC;ABL1;SRGAP3;ECT2;MARK3;CBX8;SMAD2;SMAD1;RAB4A;SMAD4;SMAD3;CBX4;SMURF2;TNK2;CBX2;ESR1;ESR2;MOB1B;COPS4;MOB1A;SP1;CDK4;MDM2;CDK1;NF2;FGFR3;YWHAE;DOCK5;HSP90AB1;WNT2B;YWHAB;DOCK9;LRRK2;PTEN;ARRB1;PTBP1;CAPZB;YWHAQ;LBR;YWHAG;GPSM3;YWHAH;DSP;HSP90AA1;CHUK;MMP3;H2AFX;PPP2R5A;TRAF1;ARAP1;RANGAP1;SMC1A;SPTB;YWHAZ;MMP9;CKAP5;CKAP4;TNFRSF1A;LATS1;SFPQ;RBL1;LATS2;MYOD1;TRAF6;ATP6V1B1;HDAC4;CAMK2B;HDAC5;RGS17;HDAC2;SRC;HDAC1;TULP3;HDAC8;HDAC9;RNF2;AURKB;MAPK8;POLR2A;RBBP4;CNR1;CCL2;PTK2B;MAPK1;NCAM1;VHL;RBBP7;HNRNPA1;MTA2;SH2B1;MAPK3;KDM4A;NOS3;MAPK14;ACTC1;APC;VAPA;VIM |
| Cellular Responses to Stimuli | 1.288^e-33^ | RB1;RPL4;ATF2;RORA;PPP2R2A;RPL8;ETS1;PSMD7;PPP2R1B;PPP2R1A;RPS18;MYC;RPS6KA1;EP300;ATP6V1E1;SKP2;TRIM21;MAP2K4;MEF2C;PRKCI;CSNK2A2;TUBA4A;AR;CCNE1;DDIT3;TNIK;SQSTM1;TP53;ATF3;ASF1A;FKBP5;ATP6V1A;VCP;CUL7;CUL3;CUL2;CUL1;FOXO3;RAI1;BAG2;UBB;BAG3;PRDX1;UBC;ABL1;HIST1H3G;PPARGC1A;CBX8;HSPA8;CREBBP;JUN;CBX4;HSPA5;ST13;HSPA4;CBX2;HSPA6;FN1;HSPA2;EIF2S2;EIF2S1;ESR1;MOV10;EIF2S3;SP1;CDK4;ALB;MDM2;CALM1;EZH2;YWHAE;CDKN1A;HSP90AB1;RPLP0;BRCA1;BMI1;IKBKB;RPS4X;TUBA1C;RPL7A;TUBA1B;MAP1LC3B;XPO1;CAPZB;NCOA2;HSP90AA1;RING1;CHUK;H2AFX;FOS;SIRT1;CCNA2;ATP6V1B1;CAMK2B;RELA;ADD1;RNF2;HSPD1;PPP2CA;MAPK9;MAPK8;TUBA3C;RXRA;RBBP4;PSMB1;RPS3;CCL2;MAPK1;VHL;RBBP7;TBL1X;MAPK3;RANBP2;SUZ12;HSPA1L;STAT1;NOS3;STAT3;MAPK14;TUBB4B;MAPK10;DNAJA1;RAD50;DNAJA4;DNAJA2;CAPZA2;CTNNB1;HDGF;HSPA1B;HSPA1A |
| Cytokine Signaling in Immune System | 2.02^e-10^ | CD86;IFITM3;ATF2;APP;EIF4A3;RORA;FGF2;SOX2;PSMD7;CCND1;PPP2R1B;PPP2R1A;MYC;RPS6KA1;TRIM21;JUNB;MAP2K4;MEF2C;HLA-B;ILF2;TUBA4A;ILF3;SQSTM1;TP53;KPNB1;CUL5;CUL1;TNFRSF11A;FOXO3;SOCS2;SOCS3;SOCS1;UBB;UBC;HIST1H3G;PLCG1;STAT5A;STAT5B;HSPA8;JUN;VCAM1;SMAD3;FN1;HSPA2;EIF2S2;EIF2S1;GH1;EIF2S3;HNRNPF;CDK1;GRB2;ARF1;CDKN1A;RPLP0;ADAR;IKBKB;TUBA1C;TUBA1B;HSP90AA1;ANXA1;CHUK;RIPK2;MMP3;PPP2R5A;IRAK3;FOS;TNFRSF1B;MMP9;YWHAZ;TNFRSF1A;PPM1B;TRAF3;TRAF6;TCP1;IRF9;CAMK2B;BECN1;SRC;RELA;PPP2CA;MAPK9;MAPK8;TUBA3C;PSMB1;CCL2;PTK2B;MAPK1;STX4;NCAM1;PAK2;SH2B1;MAPK3;SNCA;RANBP2;MAP3K3;NPM1;HSPA1L;STAT1;STAT3;MAPK14;TUBB4B;MAPK10;NEDD4;HNRNPA2B1;VIM;HSPA1B;HSPA1A |
| Chromatin Modifying Enzymes | 4.48^e-04^ | ATF2;TRRAP;PHF20;SUV39H1;IKBKAP;ING5;MECOM;CCND1;RUVBL1;WDR5;EP300;KAT7;NCOA2;PRMT5;BRD1;PRMT1;H2AFX;KAT2B;TAF6L;MORF4L1;KAT2A;BRMS1;KAT6B;KAT6A;MORF4L2;HAT1;HDAC2;MEAF6;HDAC1;HDAC8;RELA;RBBP4;NSD1;MBIP;SMYD2;HIST1H3G;TBL1X;RBBP7;MTA2;MBD3;SUZ12;SMARCE1;KDM4A;CREBBP;BRPF1;SETDB1;BRPF3;CDK4;TADA3;EZH2 |
| Adipogenesis | 1.18^e-04^ | HDAC2;HDAC1;HNRNPU;MED15;MED18;RELA;MED17;MED12;RXRA;RBBP4;MED31;EP300;RBBP7;TBL1X;PCK1;MTA2;PPARGC1A;PPARGC1B;MBD3;SMAD1;NCOA2;SREBF1;CDK19;CREBBP;SMAD4;NCOA3;MED9;NR2F2;MED26;MED29;MED25;CDK4;MED21;ZNF638 |
